# Reliable and simple spectrophotometric determination of sun protection factor: A case study using organic UV filter‐based sunscreen products

**DOI:** 10.1111/jocd.12390

**Published:** 2017-08-23

**Authors:** Soo In Yang, Shuanghui Liu, Geoffrey J Brooks, Yves Lanctot, James V Gruber

**Affiliations:** ^1^ Botaneco Inc Calgary AB Canada; ^2^ Botaneco Inc Lambertville NJ USA

**Keywords:** emulsions, oil‐in‐water, oleosomes, sun protection, sunscreens

## Abstract

**Background:**

Current in vitro SPF screening method for plant oil body (oleosome)‐based SPF products possesses significant inconsistency and low reliability in the SPF rating.

**Objectives:**

The primary objective of this study was to evaluate the reliability and reproducibility of spectrophotometrically determined sun protection factor (SPF) from oleosome‐based SPF products. The secondary objective was the data comparison of the spectrophotometric measurements against in vivo SPF testing to establish a reliable in vitro test method as a screening assay.

**Methods:**

Octyl methoxycinnamate (UVB filter) and avobenzone (UVA filter) were loaded into safflower oil bodies and formulated into oil‐in‐water emulsion‐based finished products. To evaluate the reliability between in vivo and spectrophotometric test methods, samples were dispatched to a clinical laboratory, and the reported SPF values were compared with spectrophotometric test results.

**Results:**

The observed SPF from the in vivo and spectrophotometric test results demonstrated a high correlation for SPF 30 products. Proportional correlation between the two evaluation methods was observed for SPF 15 and 50 products with slightly lesser accuracy with a smaller number of population tested in the clinical studies.

**Conclusions:**

A reliable spectrophotometric screening method for oil body‐based SPF formulas has been developed using two broadly used organic UV sunscreen actives as a case study. The results demonstrated a high level of reproducibility and reliability compared to the US FDA‐guided in vivo SPF testing method.

## 1 INTRODUCTION

Everyone on the planet is naturally exposed to sun light. Among the electromagnetic spectra from the sun, UV radiation has the highest energy among the ground‐reaching radiation. The public are more exposed to harmful UV radiation during the summer season or in tropical areas.^1^ UV radiation is also known as a cause of cutaneous malignant melanoma and is responsible for some of the fastest growing cancer cases.^1^ UV radiation has a tendency of accumulating the adverse effects over time, and noticeable changes are observed on the skin through accelerated aging processes.^2^ UV radiation induces a malfunction of the body's protective systems, such as increased inflammation, decreased tissue repair functions, and serious damage to cutaneous DNA, which elevates the occurrence of skin cancer.^3^ UV radiation damages on the skin are race, skin type, and climate dependent with, for example, the highest development rate of cutaneous malignant melanoma occurring in Australia compared to other geographic regions.^4^ In the general public, skin cancer accounts for approximately 40% of all cancers, comprising basal skin cancer (80%), squamous cell carcinomas (16%), and melanomas (4%).^5^, ^6^

The mode of UV‐induced destructive actions is governed by dosages that are higher than the tolerance limits of the body.^7^ UVB irradiation induces the formation of reactive oxygen species (ROS) in the skin and increases the oxidative stress on the skin's important macromolecules, inducing destructive damages and loss of cellular function. In addition, UVB induces more fundamental and serious damages: genetic mutation such as pyrimidine dimer formation, inflammation, and skin cancer.^5^ UVA is a deeper penetrating spectral range of UV radiation compared to UVB and is also believed to induce an increased level of oxidative stress in skin tissue. The dermis is the most sensitive area for UVA irradiation damage. Among UVA absorbing tissues and molecules, collagen is the most sensitive tissue to UVA radiation in the dermis and it causes wrinkle formations on the skin or aging effects. The mode of action has been postulated to be direct cellular damage, formation of oxygen radicals, and a reduction of antioxidant defense mechanisms, all of which cause damage to the skin.^8^

It is recommended that people wear sunscreen products as well as physical protection such as using protective clothing regularly to prevent damage to the skin. Better protection through higher sun protection factor (SPF)‐labeled products is commonly believed to provide enhanced protective benefits.^9^ A thorough evaluation of the data collected from a cohort study over 147,900 people conducted in Norway between 1991 and 2007 demonstrated the beneficial effects of using higher SPF products (ie >SPF 15) over lower SPF products (ie SPF≤15) with respect to melanoma risk.^10^ Nevertheless, a lack of public awareness on how to use sunscreen products is one of major problems facing their effective and regular use. As a result, regulatory bodies throughout the world have put more pressure on product labeling and SPF testing instructions, for example US FDA and Health Canada sunscreen product rules.^11^, ^12^

With the recent stricter regulations on SPF products in the market for both labeling and testing methods, the demand for an economical and reliable means to determine SPF from the research and development stage has increased significantly. Despite efforts to develop a reliable and compatible in vitro SPF test method, inevitable human errors and technical challenges have posed a hurdle to development of a reliable testing method. In addition to technical challenges, the cost and complexity of currently available spreading methods are obstacles in the in vitro SPF determination. This study will report a simple and readily available spectrophotometric method for SPF determination that can support fast screening and evaluation of approximate SPF values for sunscreen products.

A spectrophotometric method using organic UV filter‐containing plant oil body (oleosome)‐based products has been developed. The oleosomes are composed of individual oil droplets and the phospholipid with anchored structural surface proteins, oleosins. As the oleosome surface is covered with unique amphiphilic oleosin proteins with the hydrophobic conserved domain of the protein at the triglyceride core, the oleosome possesses unique emulsifying capability.^13^ We used purified safflower seed oil bodies in approximately 35% water emulsion. This methodological development was aimed to provide a fast, cost‐efficient, but reliable tool to screen and estimate the SPF values of sunscreen products. To build a more applicable SPF data pool, we used the most popular SPF label claim, SPF 30 products, as a model system. The spectrophotometric observation was compared to a 2‐subject in vivo clinical measurement to correlate the two data sets and to draw better comparison to real application cases. The 2‐person assays were further supported by using a 10‐person in vivo SPF study to fully validate the results of the 2‐person and spectrophotometric study results.

## 2 MATERIALS AND METHODS

### 2.1 Chemicals and equipment

*Carthamus tinctorius* (safflower) oleosomes (and) *C. tinctorius* (safflower) seedcake extract, an engineered UV active delivery system (CapSol ™; Botaneco Inc, Calgary, Canada), octyl methoxycinnamate (OMC; Escalol ™ 557; Ashland Inc, ON, Canada), butyl methoxydibenzoylmethane (Avobenzone, AVO, Parsol ™ 1789; DSM Nutritional Products, AB, Canada), dipropylene glycol dibenzoate (Finsolv ™ PG‐22; Innospec Performance Chemicals, NC, USA), fragrance (formula number: 2003BA041191; Cosmo International Fragrances, FL, USA), propylene glycol (and) diazolidinyl urea (and) iodopropynyl butylcarbamate (Liquid Germall ™ Plus; Ashland Inc, ON, Canada), and ammonium acryloyldimethyltaurate/VP copolymer (Aristoflex AVC; Clariant Personal Care, NC, USA) were purchased. Absolute ethanol was purchased from GreenField Specialty Alcohol (ON, Canada), and 40% ethanol (v/v) was prepared. Overhead mixers (Caframo stirrer BDC 1850, ON, Canada), hot plate (Cat # 97042‐738), and pH adjusting chemicals were purchased from VWR international (AB, Canada). Spectrophotometric determination of UV absorbance was carried out in 1 cm path length cuvette (Cat # 10037‐462 and ‐472, VWR international, AB, Canada), using VWR UV‐1600PC model spectrophotometer (AB, Canada).

### 2.2 Preparation of sunscreen product formulation

The formulation protocols varied depending on the target SPF levels desired, with a typical standard method summarized below. All the formulation samples were prepared in duplicate. The SPF 15, 30, and 50 formulated products were prepared as shown in Table 1.

**Table 1:** Formulation for SPF 15, 30, and 50 +^a^

	SPF 15	SPF 30	SPF 50
Phase A			
Safflower oleosome emulsion	11.70	11.70	11.70
Octyl methoxycinnamate (OMC)	1.00	2.00	7.50
Phase B			
Butyl methoxydibenzoylmethane	0.50	0.75	2.50
Dipropylene glycol dibenzoate	2.70	2.70	5.40
Phase C			
Water	81.30	80.05	70.10
Phase D			
Fragrance	0.30	0.30	0.30
Propylene glycol (and) diazolidinyl urea (and) iodopropynyl butylcarbamate	1.00	1.00	1.00
Phase E			
Ammonium acryloyldimethyltaurate/VP copolymer	1.50	1.50	1.50
Total	100.00	100.00	100.00

a The units are shown in percentage.

- Place the required amount of safflower oleosome emulsion into a 500‐mL beaker.
- Add octyl methoxycinnamate and mix at 400 rpm for 20 minutes. This is Phase A.
- Prepare Phase B at 40‐50°C with gentle mixing in a separate beaker and then add Phase B into Phase A when it reaches to room temperature (23 ± 3°C).
- Add Phase C and increase the mixing speed to 700‐750 rpm.
- Add Phase D and continue mixing for a total of 20 minutes.
- Add Phase E and decrease the mixing speed to 100‐140 rpm for 30 minutes.
- Adjust the pH to pH 5‐6 with 10 N NaOH and continue mixing for a total of 40 minutes.

After the formulation was complete in duplicate, it was stored at room temperature for at least 24 hours to allow the emulsion to stabilize, and, all the samples were evaluated using spectrophotometric methods within 30 days of sample preparation. UV absorbance from 320 to 290 nm was continuously recorded with a 5‐nm step size. The spectrophotometric SPF was calculated shown in *calculation of spectrophotometric SPF* section. One hundred gram of each sample was also sent to AMA laboratories Inc, AMA Research Laboratories Inc, New City, NY, USA, for the 2‐person and 10‐person in vivo SPF determinations.

### 2.3 Calculation of spectrophotometric SPF

The Equation (1) to determine the SPF values spectrophotometrically was modified from the work done by Mansur et al.^14^ For the determination of SPF 50, the numerical correction factor 6.65 needs to be removed to agree with the reported in vivo results. This has been determined to minimize the deviation between the calculated mean values and the in vivo results.

(1) $$\text{SPF spectro}=\{69\;\times \sum_{290\;\mathrm{nm}}^{320\;\mathrm{nm}}\left(\mathrm{EE}\;\left(\lambda \right)\;\times \;I\;\left(\lambda \right)\;\times \;\mathrm{Abs}\;\left(\lambda \right)\right)\}+6.65$$

In the equation, EE (λ), I (λ), and Abs (λ) are erythemal action spectrum, solar intensity spectrum, and UV absorbance of the sample, respectively. The EE (λ) and I (λ) are given as constants as reported and shown in Table 2.^15^

**Table 2:** EE (λ) and I (λ) values used for SPF calculation^a^

Wavelength (λ, nm)	EE (λ) × I (λ)
290	0.0150
295	0.0817
300	0.2874
305	0.3278
310	0.1864
315	0.0839
320	0.0180

a EE (λ) and I (λ) are erythemal action spectrum, solar intensity spectrum, respectively. The EE (λ) and I (λ) are given as constants.

### 2.4 Sample preparation for spectrophotometric SPF measurement

The spectrophotometric sample preparation was modified from previous work in the following ways.^16^ One hundred micrograms of SPF product was transferred into 10 mL of 40% ethanol and followed by vigorous vortexing. A visual inspection of the homogeneous sample dispersion without any aggregation was confirmed before further processing was conducted. 500 μL from the prepared sample solution was transferred using a pipette into 4.5 mL of 40% ethanol. The second solution was gently mixed by pipetting, and a 1 mL aliquot was transferred into 4 mL of 40% ethanol, followed again by a gentle pipet mixing. The prepared samples were scanned within 20 minutes of the preparation.

### 2.5 In vivo SPF determination

The in vivo tests were carried out at AMA Laboratories, New City, NY, USA, on 2‐subject panels as instructed in the 2011 US Food and Drug Administration (FDA) final rule for the sunscreen drug products.^11^ The average SPF values were quoted for comparison against the spectrophotometric SPF results. In addition, the full 10‐person study was also conducted at AMA Laboratories. The phototypes of panelists were selected based on skin types I‐III according to FDA monograph.^11^

### 2.6 Statistical analysis

The statistical processing of the data was carried out with one‐way analysis of variance (ANOVA), Student's *t* test and *P*‐value, using the package available in Microsoft Excel program.

## 3 RESULTS AND DISCUSSION

### 3.1 SPF 30 product evaluation

The investigation began using an SPF 30 product formulation as a model. The observed in vivo and spectrophotometric SPF values of the SPF 30 products are shown in Table 3. Twenty‐one SPF 30 samples were tested using both in vivo human clinical and spectrophotometric methods and the comparison between the two methods demonstrated a high level of reproducibility. The mean value of the in vivo SPF test showed an SPF value of 31.98 with the average standard deviation (SD) of 1.7. Likewise, the spectrophotometric method provided a very similar mean SPF value of 31.19 with average SD of 0.72. The difference between the mean SPF values for the two was 0.79 SPF units. To evaluate the statistical significance between the average SPF values of two groups, one‐way ANOVA analyzes were employed. The resultant *F* test (1.409) was lower than the critical *F*‐value (4.084), validating the null hypothesis. This demonstrates that the mean SPF values from the in vivo and spectrophotometric measurements are statistically identical, confirming that the spectrophotometric method is reliable and compatible to in vivo SPF testing results. Student's *t* test indicated a *P*‐value of 0.32 suggesting again the null hypothesis is valid. Thus, the two test methods possess a high correlation of statistically comparable data. The reliability of the in vivo SPF test method was also evaluated by increasing the population size from 2 to 10. The results showed a mean SPF value of 33.15 compared to the spectrophotometric SPF value of 33.31 as shown in Tables 4, 5, 6. This further demonstrates the high level of reliability and reproducibility of the spectrophotometric SPF method presented here.

**Table 3:** In vivo and spectrophotometric SPF results of SPF 30 products^a^

Samples	In vivo SPF	SD	In vivo Ref #	Spec SPF	SD
30‐A	32.40	4.80	O‐4959	28.60	0.96
30‐B	30.00	0.00	O‐6096	28.90	0.03
30‐C	32.25	3.18	O‐6095	29.09	0.21
30‐D	32.25	3.18	O‐6547	32.69	0.25
30‐E	32.25	3.18	O‐5567	30.06	0.78
30‐F	34.50	0.00	O‐6546	33.10	1.57
30‐G	30.00	0.00	O‐6860	32.98	0.72
30‐H	28.05	2.76	O‐6093	31.99	0.40
30‐I	32.25	3.18	O‐6545	33.50	1.83
30‐J	34.50	0.00	O‐6859	33.31	1.82
30‐K	34.50	0.00	O‐6858	31.45	0.50
30‐L	26.10	0.00	O‐6959	27.80	0.33
30‐M	30.00	0.00	O‐7421	28.53	0.12
30‐N	34.50	0.00	O‐7970	29.63	0.31
30‐O	32.25	3.18	O‐6548	32.54	2.61
30‐P	34.50	0.00	O‐6625	33.35	0.04
30‐Q	34.50	0.00	O‐6872	31.51	0.58
30‐R	32.25	3.18	O‐6549	33.27	1.64
30‐S	34.50	0.00	O‐6626	31.97	0.24
30‐T	30.00	0.00	O‐6550	30.98	1.35
30‐U	30.00	0.00	O‐6874	29.67	0.51
Mean	31.98	1.70		31.19	0.72

a The mean values of in vivo and spectrophotometric SPF test results with standard deviations (SD) are shown. “In vivo Ref #” and “Spec SPF” are the sample ID numbers from the clinical laboratory and spectrophotometric SPF, respectively.

**Table 4:** Ten subject in vivo SPF result^a^

Subject/hr	MED^b^	I (Amps)	Skin Type^c^	MED I (J/M^2^)	MED II (J/M^2^)	Static SPF
A	126.4	6.20	II	28.44	28.44	34.50
B	126.9	6.00	III	55.55	55.55	34.50
C	127.6	6.50	II	28.44	28.44	34.50
D	126.2	6.00	II	35.55	35.55	34.50
E	127.9	6.20	II	28.44	28.44	34.50
F	128.3	6.50	II	55.55	55.55	34.50
G	127.5	6.10	II	28.44	28.44	30.00
H	126.4	6.20	II	35.55	35.55	30.00
I	128.3	6.00	III	55.55	55.55	34.50
J	127.9	6.20	I	28.44	28.44	30.00

a AMA reference number: O‐6859. The initial two‐subject test result of this sample is shown in 30‐J, Table 3.

b MED, minimal erythemal dose;

c Skin Type, the skin types based on Fitzpatrick Scale.

**Table 5:** Analysis of in vivo SPF result shown in Table 4, a

Mean (x)	SD	SE	SE % of mean	n	Upper 5% *t*‐dist	*A* value^b^	Label SPF
33.15	2.17	0.69	2.08	10	1.8331	1.2579	31

a Standard deviation (SD), standard error (SE), population (n), and *t*‐distribution (*t*‐dist) are shown.

b *A* value = (*t*‐dist × SD)/n^1/2.^

**Table 6:** Spectrophotometric UV absorbance for SPF determination^a^

WL (nm)	Sample 1‐1 (abs)	Sample 1‐2 (abs)	Sample 2‐1 (abs)	Sample 2‐2 (abs)
320	0.411885	0.390453	0.376699	0.389518
315	0.433045	0.410343	0.394875	0.408998
310	0.435637	0.412076	0.396323	0.411177
305	0.422165	0.397174	0.382541	0.397207
300	0.379695	0.355649	0.344159	0.354071
295	0.346860	0.322380	0.313441	0.319926
290	0.293353	0.273854	0.267074	0.265706

a UV absorbance (abs) collected on the same sample shown in Table 4 Duplicate sample preparations were carried out (sample 1 and 2) with duplicate samplings (sample number—1 and—2). UV absorbance from 320 to 290 nm was continuously recorded with a 5‐nm step size. The spectrophotometric SPF was calculated using Equation (1).

These observations are very encouraging due to low reproducibility of currently available in vitro SPF test methods.^17^ The current in vitro test methods are popular but not accepted by any regulatory bodies in the world.^18^ The rubbing on the plate type in vitro SPF methods requires intensive training and care to increase the accuracy of the results, mainly due to sample preparation and intrinsic drawbacks in the techniques. With respect to reported in vitro SPF testing methodologies, the SPF values are prone to potential experimental errors, depending on the skill levels of the operators. Even and homogeneous distribution of the sample on the substrate plate using a finger cot is another area for potential error, considering the small amount of sample applied (2 mg·cm^−2^ or various amounts suggested for the tests) prior to UV irradiation of the sample.^11^ Therefore, SPF ratings from reputable clinical laboratories are currently the sole SPF tests accepted by the regulatory bodies around the world. In this aspect, our SPF screening method for oleosome‐based formulas is an efficient means to alleviate cost and time required for the routine screening.

### 3.2 Preliminary evaluation of SPF 15 and 50 product

To expand the compatibility of the spectrophotometric SPF test method, SPF 15 and 50 formulated products were also evaluated using both in vivo and spectrophotometric testing methods (Table 7). Spectrophotometric SPF testing results of both 15 and 50 formulations showed slightly higher SPF values compared to in vivo test results. However, due to the smaller tested population size, it is premature to conclude that a confidence level comparable to the SPF 30 results reported above was achieved for the spectrophotometric method to measure SPF 15 and 50 products. However, preliminary expanded in vivo testing appears to be demonstrating good consistency among SPF 15 and 50 formulations as the data pool with the number of samples is increased (data not presented). Further work, including testing products with additional popular approved UV actives, will be initiated to further expand the scope of the spectrophotometric testing results reported here.

**Table 7:** SPF data comparison between in vivo and spectrophotometric methods^a^

ID #	In vivo SPF	SD	In vivo Ref #	Spec SPF	SD
SPF 15	16.80	1.64	O‐9529	20.81	0.33
SPF 50^b^	53.75	5.3	O‐9726	54.34	4.57

a The mean values of in vivo and spectrophotometric SPF test results with standard deviations (SD) are shown. “in vivo Ref #” and “Spec SPF” are the sample ID numbers from a clinical laboratory and spectrophotometric SPF, respectively.

b SPF was calculated without considering 6.65 correction constant shown in Equation (1) (see “Calculation of spectrophotometric SPF” section).

## References

[1] Dupont E , Gomez J , Bilodeau D . Beyond UV radiation: a skin under challenge. Int J Cosmet Sci. 2013;35:224‐232. 10.1111/ics.12036 23406155

[2] De Fabo EC , Noonan FP , Fears T , et al. Ultraviolet B but not ultraviolet a radiation initiates melanoma. Cancer Res. 2004;64:6372‐6376. 10.1158/0008-5472.CAN-04-1454 15374941

[3] Amaro‐Ortiz A , Yan B , D'Orazio JA . Ultraviolet radiation, aging and the skin: prevention of damage by topical cAMP manipulation. Molecules. 2014;19:6202‐6219. 10.3390/molecules19056202 PMC4344124 24838074

[4] Wei Q , Lee JE , Gershenwald JE , et al. Repair of UV light‐Induced DNA damage and risk of cutaneous malignant melanoma. J Natl Cancer Inst. 2004;95:308‐315. 10.1093/jnci/95.4.308 12591987

[5] Narendhirakannan RT , Hannah MAC . Oxidative stress and skin cancer: an overview. Indian J Clin Biochem. 2013;28:110‐115. 10.1007/s12291-012-0278-8 PMC3613501 24426195

[6] Einspahr JG , Stratton SP , Bowden GT , et al. Chemoprevention of human skin cancer. Crit Rev Oncol Hematol. 2002;41:269‐285. 10.1016/s1040-8428(01)00185-8 11880204

[7] Kehrer JP . Free radicals as mediators of tissue injury and disease. Crit Rev Toxicol. 1993;23:21‐48. 10.3109/10408449309104073 8471159

[8] Ou‐Yang H , Stamatas G , Kollias N . Dermal contributions to UVA‐induced oxidative stress in skin. Photodermatol Photoimmunol Photomed. 2009;25:65‐70. 10.1111/j.1600-0781.2009.00403.x 19292780

[9] Bastuji‐Garin S , Diepgen TL . Cutaneous malignant melanoma, sun exposure, and sunscreen use: epidemiological evidence. Br J Dermatol. 2002;146(Suppl 61):24‐30. 10.1046/j.1365-2133.146.s61.9.x 11966729

[10] Ghiasvand R , Weiderpass E , Green AC , et al. Sunscreen use and subsequent melanoma risk: a population‐based cohort study. J Clin Oncol. 2016;34:3976‐3983. 10.1200/JCO.2016.67.5934 27621396

[11] Department of health and human services‐ U.S. Food and Drug Administration . Labeling and effectiveness testing: sunscreen drug products for over‐the‐counter human use. 21 CFR Parts 201 and 310. Fed Regist. 2011;76:35620‐35665. 21682059

[12] Health Products and Food Branch‐Health Canada . Sunscreen monograph. 2013;2:1‐15.

[13] Tzen JTC . Integral proteins in plant oil bodies. ISRN Botany. 2012;2012:1‐16.

[14] Mansur JS , Breder M , Mansur M , et al. Determinação do fator de proteção solar por espectrofotometria. An Bras Dermatol. 1986;61:121‐124.

[15] Sayre RM , Agin PP , LeVee GJ , et al. A comparison of *in vivo* and *in vitro* testing of sunscreening formulas. Photochem Photobiol. 1979;29:559‐566. 10.1111/j.1751-1097.1979.tb07090.x 441130

[16] Dutra EA , Almança D , Kedor ERM , et al. Determination of sun protection factor (SPF) of sunscreens by ultraviolet spectrophotometry. Braz J Pharm Sci. 2004;40:381‐385.

[17] Fujikake K , Tago S , Plasson R , et al. Problems of *in vitro* SPF measurements brought about by viscous fingering generated during sunscreen applications. Skin Pharmacol Physiol. 2014;27:254‐262. 10.1159/000356298 24923467

[18] Schalka S , dos Reis VMS . Sun protection factor: meaning and controversies. An Bras Dermatol. 2011;86:507‐515. 10.1590/s0365-05962011000300013 21738968

